# Exercise, Demethylase FTO, Neurological Disorders, and Neuropathic Pain: Potential Molecular Mechanisms

**DOI:** 10.1002/cns.71051

**Published:** 2026-07-27

**Authors:** Yanan Zheng, Yili Zheng, Peijie Chen, Xueqiang Wang

**Affiliations:** ^1^ Department of Rehabilitation Medicine The First Affiliated Hospital of Anhui Medical University Hefei Anhui China; ^2^ Department of Sport Rehabilitation Shanghai University of Sport Shanghai China; ^3^ Department of Rehabilitation Medicine The Second Affiliated Hospital of Wenzhou Medical University Wenzhou Zhejiang China

**Keywords:** exercise, FTO, N6‐methyladenosine, neurological disorders, neuropathic pain, review

## Abstract

**Main Problem and Methods:**

This review aims to summarize the role of fat mass and obesity‐associated protein (FTO) in neurological disorders and neuropathic pain (NP).

**Results:**

Key findings suggest that in neurological disorders such as Alzheimer's disease, Parkinson's disease, and depression, FTO‐regulated m^6^A modification plays a critical role in the hippocampus and striatum. FTO‐mediated m^6^A modification is involved in the pathological processes of NP. These findings suggest that FTO may serve as a potential therapeutic target for neurological disorders and NP. Common mechanisms may include the regulation of downstream mTOR and BDNF/TrkB signaling pathways, as well as modulation of neuronal excitability and synaptic plasticity. However, research on the relationship between exercise and m^6^A modification remains in its early stages. Emerging evidence suggests that exercise reduces FTO expression and increases m^6^A levels in the hippocampus and hypothalamus, indicating that exercise may serve as an effective intervention for modulating epigenetic modifications in the central nervous system.

**Conclusions:**

This implies that exercise may serve as an effective intervention for modulating epigenetic modifications, potentially by downregulating the demethylase FTO, regulating m^6^A modification, enhancing synaptic plasticity, modulating neuronal excitability, and providing neuroprotection, thereby contributing to disease mitigation. We hypothesize that exercise may regulate neurological disorders and NP through FTO‐mediated m^6^A modification, with FTO potentially serving as a biomarker.

## Graphical abstract

This review summarizes the role of FTO‐regulated m^6^A modification in Alzheimer's disease, Parkinson's disease, depression, and neuropathic pain. Exercise may ameliorate neural plasticity and neuronal excitability by downregulating FTO and enhancing m^6^A modification. We propose that FTO could serve as a potential epigenetic target through which exercise relieves neurological disorders and neuropathic pain.

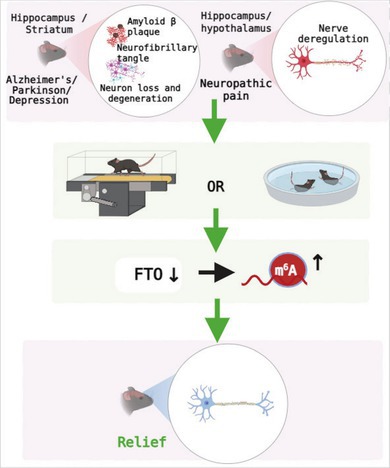

## 1 Introduction

Neurodegenerative diseases such as Alzheimer's disease (AD) and Parkinson's disease (PD), along with other nervous system‐related disorders including depression and neuropathic pain (NP), constitute major contributors to global mortality and disability [1, 2]. They are also among the most widely concerned neurological disorders of complex etiology in clinical practice. Recent studies have highlighted the role of N6‐methyladenosine (m^6^A) RNA modification in neurological disorders and NP. As the most common and abundant RNA methylation modification in mammals, m^6^A is predominantly located at RRACH motifs and 3′ untranslated regions (3′‐UTR) [3, 4, 5]. It regulates RNA metabolism, including transcription, splicing, and translation, thereby influencing gene expression and playing a crucial role in neuronal metabolism, myelination, learning, and memory [6, 7]. The dynamic regulation of m^6^A levels is mediated by “writers” (e.g., METTL3/14), “erasers” (e.g., Fat mass and obesity‐associated protein (FTO)/ALKBH5), and “readers” (e.g., YTHDF family proteins) [3, 4]. Recent research found that the dysregulation of demethylase FTO‐regulated m^6^A was closely associated with the onset and progression of AD [8, 9, 10], PD [11], Depression [12], and NP [13, 14, 15]. These findings revealed the potential role of FTO in neurological disorders and NP.

As a positive lifestyle intervention, exercise significantly improves neurological health. It enhances neurogenesis, improves cognitive function, suppresses inflammatory responses, and strengthens neuroprotective mechanisms, thereby delaying progression of neurological disorders [16, 17, 18]. Emerging evidence suggests that exercise alleviates heart failure through FTO‐regulated m^6^A modification, and exercise decreases FTO expression while increasing m^6^A levels in the hippocampus and hypothalamus [19, 20]. This suggests that exercise may serve as an effective intervention for modulating epigenetic modification, potentially by downregulating the demethylase FTO and regulating m^6^A methylation, thereby exerting effects in disease amelioration. However, research on the relationship between exercise and m^6^A modification is still in its early stages. Our recent preclinical study demonstrated that swimming exercise effectively alleviated mechanical and cold allodynia in a spared nerve injury (SNI) mouse model of NP [21]. It must be acknowledged that direct evidence linking exercise to m^6^A modification in neurological disorders and NP is lacking. This analgesic effect was mechanistically linked to the downregulation of FTO and a concomitant increase in m^6^A methylation of the microRNA miR‐183 within the dorsal root ganglia, leading to the suppression of downstream pain‐related targets. Accordingly, this review aims to summarize the role of FTO in neurological disorders (with a focus on AD, PD, and depression) and NP, analyze the effects of exercise on FTO‐mediated m^6^A modification in neurological and related diseases, and summarize underlying mechanisms.

## 2 m^6^A and FTO

### 2.1 Molecular Mechanisms of m^6^A Modification

Research on epigenetic mechanisms in neurological disorders is advancing, and m^6^A modification has emerged as a key area of study. m^6^A modification refers to the process by which a methyl group is selectively added to the N6 position of adenosine under the action of the RNA methyltransferase complex [22]. This modification typically occurs in the RRACH motif (R = A/G, H = A/C/U), particularly around transcription start sites and near stop codons in the 3′‐UTR [3, 4, 23]. It is widely present in mRNA, long noncoding RNAs (lncRNAs) [24], microRNAs (miRNAs) [25], small nuclear RNAs (snRNAs) [26], small nucleolar RNAs (snoRNAs) [27], and ribosomal RNAs (rRNAs) [28, 29], covering nearly the entire transcriptome. Dysregulation of m^6^A modification is closely associated with various physiological defects and neurological disorders [13, 14, 30, 31, 32, 33]. Recent research has focused on identifying m^6^A modification sites and constructing modification profiles through methylated RNA immunoprecipitation sequencing to elucidate its transcriptional regulatory mechanisms in diseases [3, 34]. In‐depth research on the association between m^6^A modification and neurological disorders and NP may provide new targets and insights for diagnosis and treatment.

### 2.2 m^6^A Demethylase FTO

FTO is a key m^6^A demethylase [35, 36]. Studies show that FTO knockdown increases m^6^A levels in mRNA, whereas FTO overexpression reduces m^6^A levels [37]. FTO is also involved in nuclear RNA processing events, including mRNA translation, splicing, and metabolism. Through its m^6^A demethylation function, FTO participates in nuclear RNA processing events, including mRNA translation, splicing, and metabolism [38, 39, 40, 41, 42, 43, 44, 45]. FTO and ALKBH5 belong to the AlkB dioxygenase family, which relies on α‐ketoglutarate and Fe (II) to catalyze m^6^A demethylation [40]. FTO's primary role in m^6^A demethylation is widely recognized, despite some studies suggesting a preference for m^6^Am [46, 47]. FTO was first identified as an obesity susceptibility gene [48]. Subsequent studies have shown its role in regulating energy metabolism in adipose and muscle tissues through m^6^A demethylation, influencing obesity, mitochondrial metabolism, and cardiomyocyte contractility [40]. Dysregulation of FTO expression in the hypothalamus is closely associated with obesity and type 2 diabetes [49, 50]. Mice with suppressed FTO expression exhibit growth retardation and reduced adipose tissue [51, 52].

Current research on FTO primarily involves cancer, neurological disorders, cardiovascular diseases, and osteoarthritis. In the central nervous system, FTO is highly expressed and closely associated with neuronal excitability, stress responses, and neurogenesis [13, 31, 53]. After peripheral nerve injury, FTO influences the expression of NP‐related genes in the injured DRG through its demethylation function, contributing to NP development and progression [33, 54, 55, 56]. In summary, the m^6^A modification dynamically and reversibly regulates gene expression, and the demethylase FTO plays an important role in neural function. FTO may be a target for the treatment and rehabilitation of neurological disorders and neuropathies.

## 3 Mechanisms of FTO‐Regulated m^6^A Modification in Neurological Disorders and NP

### 3.1 Role of FTO in Alzheimer's Disease (AD)

AD is a common neurodegenerative disorder characterized by the accumulation of β‐amyloid (Aβ) and the abnormal phosphorylation of Tau protein [57, 58]. Li et al. [9] reported elevated FTO levels in the brains of 3xTg (triple transgenic)‐AD mice, where FTO targets TSC1 to activate mTOR signaling, promoting Tau phosphorylation and contributing to AD pathology. Defects in insulin signaling in humans and mice with diabetes and obesity activate FTO in brain tissue. Conditionally knocking out FTO in neurons reduces cognitive deficits in 3xTg‐AD mice [9]. Reducing FTO expression reverses abnormal Tau phosphorylation and alleviates cognitive deficits caused by Aβ aggregation [10]. Additionally, Han et al. observed increased m^6^A methylation in the cortex and hippocampus of amyloid precursor protein (APP)/presenilin‐1 (PS1) transgenic AD mice (exhibiting accelerated Aβ deposition), along with increased expression of the methyltransferase METTL3 and decreased expression of the demethylase FTO [8]. Peaks of significant change are concentrated in synaptic growth at neuromuscular junctions, snRNA transcription, and smooth signaling pathways involved in dorsal‐ventral neural tube modeling [8]. Interestingly, m^6^A modifications are time‐specific during brain development and aging [46, 48]. Shafik et al. [59] found that m^6^A peaks decline during brain maturation but increase with aging; the differential m^6^A peaks are enriched in the alternative untranslated regions of genes that influence age‐related pathways. Additionally, this study found that METTL3 levels decreased slightly, whereas FTO levels increased [59]. It is noted that Li et al. [9] and Shafik et al. [59] found the expression of FTO increases in AD, but Han et al. [8] showed the expression of FTO decreases. The reasons for the difference may be attributed to multiple factors. First, the two AD mouse models differ in genetic background, pathological characteristics and disease progression timelines. For instance, 3xTg‐AD mice exhibit both Aβ and tau pathologies, whereas APP/PS1 mice predominantly feature Aβ deposition. Second, FTO expression may vary with disease stage, and variations in tissue sampling time points across studies could influence the results. Shafik et al. [59] found a progressive upregulation of FTO with aging. Moreover, in brain tissues from patients with Alzheimer's disease, FTO expression may correlate with Braak staging, although systematic longitudinal studies are currently lacking [60]. Collectively, these factors suggest that the regulation of FTO in AD is complex, highlighting the need for future studies to integrate multiple models, time points, and detection approaches.

Furthermore, FTO regulates neurogenesis through multiple pathways, including the expression of BDNF signaling, platelet‐derived growth factor receptor, and cytokine signaling inhibitor 5, as well as adenosine levels [61, 62, 63]. FTO modulates memory function through the BDNF/TrkB pathway. It increases the m^6^A modification of Bdnf mRNA and promotes neuronal apoptosis following aluminum‐induced oxidative stress [62]. Prolonged light exposure inhibits FTO, increases the m^6^A modification of Trkb mRNA, and promotes its degradation, suppressing the BDNF/TrkB/ERK pathway and impairing cognition [64]. Compared with naïve mice, the specific loss of FTO results in neurogenetic abnormalities, inhibits neuronal development, and reduces brain volume and brain weight. The outcomes include reduced proliferation and differentiation of Neural Stem Cells (NSCs) in adulthood, as well as impaired memory and learning function [31, 65, 66].

In the synaptic transcriptome, m^6^A modification is abundant and inversely correlated with synaptic RNA transcriptional abundance, suggesting its role in regulating synaptic function via localized mRNA degradation [32]. Highly methylated transcripts are linked to synaptic development and disorders like intellectual disability, autism, and schizophrenia [32]. Vitamin B12 deficiency reduces S‐adenosylmethionine (SAM) synthesis, creating a hypomethylation environment that may secondarily upregulate FTO expression as a compensatory response, thereby potentially lowering overall methylation levels [67]. Although hyper‐methylating protein kinase C mRNA, thereby impairing brain development and neuroplasticity [67]. FTO, enriched in neuronal dendrites and synapses, is targeted by inhibitors that suppress axonal elongation through the regulation of GAP‐43 [42, 61]. An FTO inhibitor, Rhein, enhances the m^6^A modification of GAP‐43 mRNA, inhibiting axonal elongation and improving cognitive deficits in AD models [42, 68]. Another FTO inhibitor, MO‐I‐500, alleviates AD‐related pathology by reducing oxidative stress and metabolic dysfunction in astrocytes [69].

**FIGURE 1:**
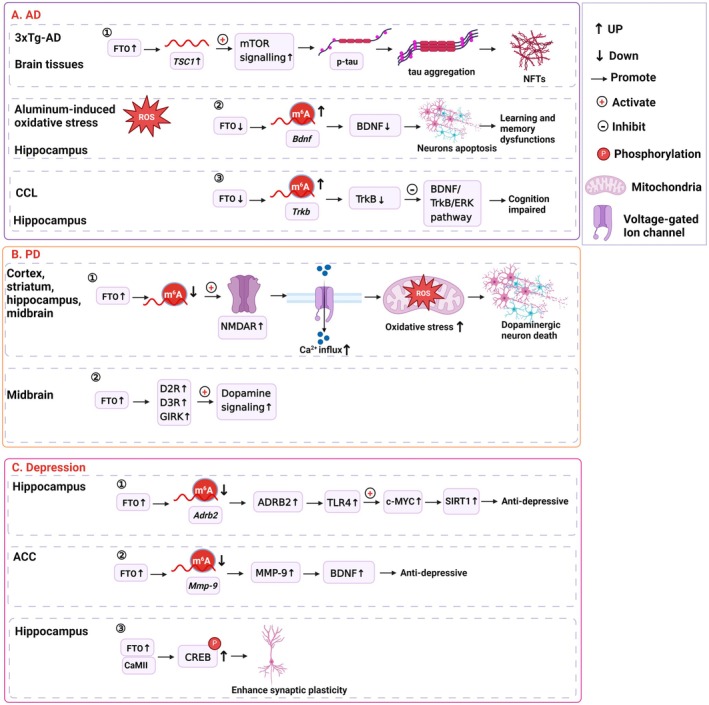
Role of FTO‐regulated m^6^A modification in neurological disorders. (A) AD. (B) PD. (C) Depression. 3xTg, the triple transgenic; AD, Alzheimer disease; BDNF, brain‐derived neurotrophic factor; ERK, extracellular signal‐regulated kinase; FTO, fat mass and obesity‐associated protein; m^6^A, N6‐methyladenosine; mTOR, mammalian target of rapamycin; NFT, neurofibrillary tangles; NMDAR, N‐methyl‐D‐aspartate receptor; PD, Parkinson's disease; p‐tau, phosphorylated tau; ROS, reactive oxygen species; TrkB, tropomyosin receptor kinase B; TSC1, tuberous sclerosis complex. The direction arrows “↑” indicates upregulation and “↓” indicates downregulation.

In summary, increased FTO activates mTOR signaling in the AD mice model, leading to abnormal Tau phosphorylation. Reducing FTO expression reverses these effects, highlighting its role in neurogenesis and memory regulation. Knocking down the Fto gene in naïve mice leads to abnormal neurogenesis, inhibits neuronal development, and impairs neuronal differentiation, also affecting the processing of BDNF in the hippocampus and resulting in impaired memory and learning (Figure 1 and Table 1).

**TABLE 1:** Effects of FTO on AD, PD, and depression.

Species/age/sex/strain	Model/disease	m^6^A regulatory factor	Behavioral test	Sample	Main results	References
Mice; 8‐week‐old; female to male: 2:1; C57BL/6J	CCL; depression	FTO	MWMT	Hippocampus	Inhibiting FTO increase m^6^A modification of *Trkb* mRNA, promote its degradation, inhibit BDNF/TrkB/ERK pathway, and impair cognition	Yang et al. [64]
Mice; 4–6 weeks old; male; C57BL/6J	UCMS; CRS; SDS; depression	FTO	FST; TST; SCT; SPT; SIT; NSFT; OFT; EZM	Hippocampus	FTO decreased. Inhibition of *Fto* expression led to depression‐like behavior, whereas overexpression of *Fto* reduce m^6^A modification of ADRB2, increase its expression and activate the c‐MYC/SIRT1 pathway, thus relieve depressive episodes	Liu et al. [70]
Mice; adult; male; wide‐type	3xTg‐AD mice; AD	FTO	NA	Brain tissues, primary neurons cells	Increased FTO target TSC1, activate mTOR signaling, and promote Tau phosphorylation	Li et al. [9]
Rats; 2‐month‐old; male; SD	Al(mal)3‐induced oxidative stress model; AD	FTO	Water maze experiment	Hippocampus	FTO decreased, m^6^A modification of *Bdnf* mRNA increased, promoting neuronal apoptosis and impaired learning and memory function	Song et al. [62]
Rats; male; SD	6‐OHDA; PD	FTO	The number of rotations	Cortex, striatum, hippocampus, and midbrain	FTO increased, promote m^6^A demethylation, activate NMDAR, induce oxidative stress and mitochondrial dysfunction, lead to dopaminergic neuron death	Chen et al. [11]

Abbreviations: 3xTg‐AD, the triple transgenic Alzheimer disease; Al(mal), aluminum‐maltolate; BDNF, brain‐derived neurotrophic factor; CCL, chronic constant light exposure; CRS, Chronic restraint stress; ERK, extracellular signal‐regulated kinase; EZM, elevated zero maze test; FST, forced swimming test; FTO, fat‐mass and obesity‐associated proteins; m^6^A, N6‐methyladenosine; mTOR, mammalian target of rapamycin; MWMT, Morris water maze test; NA, not applicable; NMDAR, N‐methyl‐D‐aspartate receptor; NSFT, novel suppressed feeding test; OFT, open field test; PD, Parkinson's disease; SCT, sucrose consumption test; SD, Sprague–Dawley; SDS, social defeat stress; SIT, social interaction test; SPT, sucrose preference test; TrkB, tropomyosin receptor kinase B; TSC1, tuberous sclerosis complex; TST, tail suspension test; UCMS, unpredictable chronic mild stress.

### 3.2 Role of FTO in Parkinson's Disease (PD)

PD, the second most common neurodegenerative disorder, involves m^6^A modification in dopaminergic signaling [71]. In PD rat models, striatal m^6^A levels are reduced, and FTO levels are elevated. FTO overexpression or the use of m^6^A inhibitors, such as cycloleucine, promotes m^6^A demethylation, which activates NMDA receptors and induces oxidative stress and mitochondrial dysfunction, ultimately leading to the death of dopaminergic neurons [11]. Moreover, FTO knockdown increases m^6^A levels and reduces apoptosis, suggesting its therapeutic potential [11]. The potential mechanism of oxidative stress in this study is that the overexpression of FTO increases the content of reactive oxygen species in mitochondrial respiratory complex II, promoting the oxidation of lipids, proteins, and DNA, thereby exacerbating mitochondrial damage and dopaminergic neuronal cell injury [11]. Simultaneously, intracellular Ca^2+^ overload also aggravates this damage [11]. Additionally, the expression of the anti‐apoptotic B‐cell lymphoma‐2 (Bcl‐2) protein in the cytoplasm is reduced, whereas the expression of Bcl‐2‐associated X protein (Bax) increases [11, 72]. Apoptosis‐inducing signals can trigger conformational changes in Bax, facilitating its integration into the outer mitochondrial membrane and ultimately inducing apoptosis. On one hand, it reveals that FTO overexpression may induce dopaminergic neuronal apoptosis by triggering the mitochondrial apoptotic pathway. On the other hand, it may lead to dopaminergic neuronal death by inducing apoptosis in striatal cells [11]. Supporting this, FTO knockdown can increase m^6^A levels and reduce apoptosis in dopaminergic neurons in vitro PD cell models [11, 73, 74].

Manganese (Mn) exposure, a major environmental risk factor for PD, reduces FTO expression. Overexpression of FTO could downregulate the methylation of ephrin‐A5 and ephrin‐B2 m^6^A, thereby improving motor dysfunction and reducing Mn‐induced cytotoxicity [75]. Hess et al. found that FTO disrupts dopamine receptors by targeting proteins such as GNAO1 and GRIN1, thereby activating D2R‐D3R‐GIRK channels and promoting dopamine signaling [53]. Selberg et al. [74] demonstrated that FTO inhibitors exhibit neuroprotective effects in PD models, with FTO inhibitors being more effective than ALKBH5 inhibitors. Entacapone, an FTO inhibitor, binds to FTO and promotes the m^6^A modification of FOXO1, thereby regulating metabolic pathways and suggesting its potential for PD treatment [31].

The above research indicates that FTO may primarily impact PD's dopamine (DA) pathway. It induces oxidative stress and Ca^2+^ influx in dopamine neurons, impairs mitochondrial function, triggers apoptosis in striatal cells, and promotes the death of dopamine neurons (Figure 1 and Table 1).

### 3.3 Role of FTO in Depression

Liu et al. found that the expression of FTO is downregulated in the hippocampus of depressed patients and mouse models of depression. FTO inhibition leads to depression‐like behavior in adult mice. Furthermore, FTO overexpression reduces the m^6^A modification of ADRB2, thereby increasing its expression and activating the c‐MYC/SIRT1 pathway, which inhibits depressive episodes [12]. Shen et al. observed that hippocampal FTO overexpression enhances synaptic plasticity by interacting with CaMKII and increasing CREB phosphorylation, thereby restoring dendritic structure in depression models [76]. Additionally, peripheral nerve injury reduces binding of FTO and MMP‐9 mRNA, increasing m^6^A levels and lowering pro‐BDNF and m‐BDNF levels in the anterior cingulate cortex, leading to anxiety and depression‐like behaviors [77, 78]. In summary, m^6^A plays a significant role in depression, and a high expression of the FTO gene is associated with the alleviation of depressive symptoms. FTO exerts antidepressant effects by promoting synaptic plasticity and restoring dendritic structures (Figure 1 and Table 1).

### 3.4 Role of FTO in Neuropathic Pain (NP)

NP was characterized by hyperalgesia, allodynia, and spontaneous pain arising from damage to the somatosensory nervous system, affecting 6.9%–10% of the global population [79, 80, 81]. The pathogenesis of NP involves peripheral and central sensitization, dysregulated epigenetic modifications, ion channel dysfunction, and neuroimmune activation [2]. Recent studies found that FTO regulates NP‐related gene expression, such as ion channels and neurotransmitter receptors, through m^6^A modification [13, 14, 15, 33, 54, 55, 56]. After peripheral nerve injury, FTO expression increases in the DRG, reducing the m^6^A modification of Ehmt2 mRNA, increasing G9a protein expression, suppressing μ‐opioid receptor (MOR) transcription, and enhancing neuronal excitability [33]. This research also found that FTO gene activation is associated explicitly with nerve injury. Overexpressing FTO in uninjured DRG neurons induces NP‐like symptoms [33]. Knocking down FTO reverses the loss of m^6^A modification sites of Ehmt2 mRNA, restoring opioid receptor expression and alleviating NP [33]. FTO inhibitors (e.g., meclofenamic acid and N‐CDPCB) alleviate NP symptoms by blocking the upregulation of G9a and restoring the expression of MOR [82, 83].

FTO‐regulated m^6^A modification also modulates other NP‐related genes. For example, FTO binds to Mmp24 mRNA, reducing its m^6^A modification and enhancing translation, which promotes ERK phosphorylation and NP [84]. FTO is not limited to its role in peripheral neurons; new discoveries have also been made about its role in pain brain circuits. In thalamic hemorrhage models, FTO upregulates the expression of TLR4, contributing to the occurrence and maintenance of allodynia by stabilizing the loss of the m^6^A site in Tlr4 mRNA of thalamic neurons [85]. METTL3, an m^6^A methyltransferase, also plays a role in NP. Downregulating METTL3 increases m^6^A levels and the stability of Tet1 mRNA, leading to hyperalgesia, whereas METTL3 overexpression reverses this effect [86]. In SNI rat models, METTL3 mediates the m^6^A methylation of pri‐miR‐150, promoting miR‐150 maturation and targeting Bdnf mRNA [87].

**FIGURE 2:**
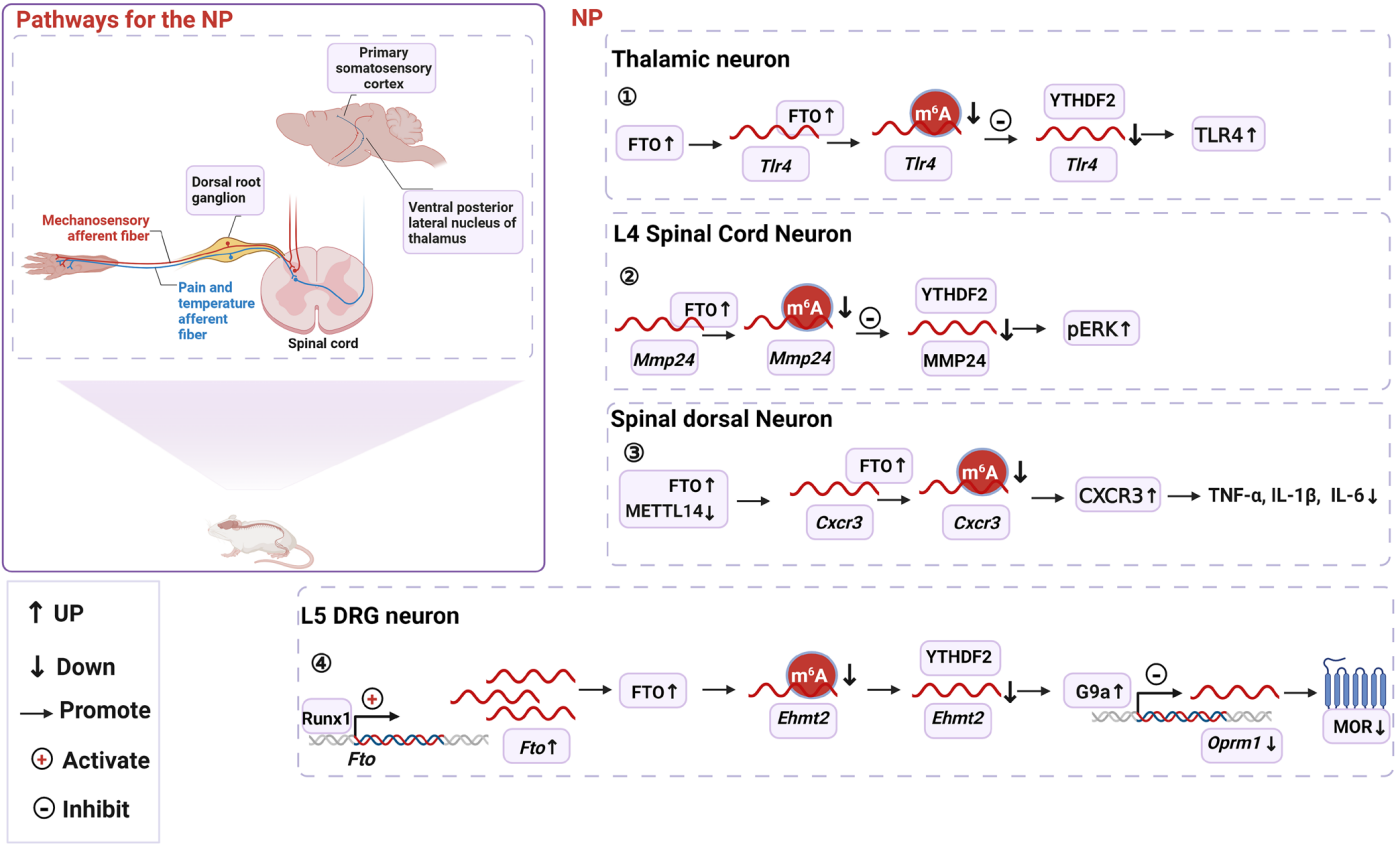
Role of FTO‐regulated m^6^A modification in neuropathic pain. CCL, chronic constant light exposure; DRG, dorsal root ganglia; ERK, extracellular signal‐regulated kinase; FTO, fat mass and obesity‐associated protein; IL, interleukin; m^6^A, N6‐methyladenosine; MOR, μ opioid receptors; NP, neuropathic pain; TNF‐α, tumor necrosis factor‐α.

Beyond m^6^A modification, epigenetic mechanisms such as DNA methylation and histone modifications also play crucial roles in NP by altering gene expression. The DNA methylation pattern in the prefrontal cortex of SNI mice exhibits time‐specific changes following nerve injury, which in turn influence pain‐related gene expression [70, 88, 89]. In SNI rats, TET1 expression increases in the prefrontal cortex, whereas DNMT1 expression increases in the hippocampus [70, 89]. In the PSNL mouse model, reduced DNA methylation in the prefrontal cortex and periaqueductal gray is associated with mechanical and cold allodynia [90]. Human genome‐wide analyses reveal distinct DNA methylation patterns in the blood cells of NP patients compared with those of healthy controls and individuals with nociceptive pain [91]. In the SNL rat model, increased TET1 expression in the ipsilateral spinal dorsal horn results in BDNF promoter demethylation, thereby elevating BDNF levels and contributing to spinal sensitization in pain pathways [92]. TET1 knockout reduces these changes. Initially, DNMT enzymes promote BDNF promoter methylation, but this process shifts as TET1 expression increases. Above research on epigenetic mechanisms in NP reveals the critical role of DNA methylation and histone modifications in pain regulation, offering a theoretical basis for developing novel analgesics [70, 88, 89, 93]. In summary, the demethylase FTO‐regulated m^6^A modification modulates pain‐related gene expression, playing an important role in NP development and maintenance, with its inhibitors showing therapeutic potential (Figure 2 and Table 2).

**TABLE 2:** Effects of FTO on NP.

Species/age/sex/strain	Model	m^6^A regulatory factor	Behavioral test	Sample	Main results	References
Mice; 6–8 weeks old; male; C57BL/6J	CCI	FTO	Mechanical and heat tests	ACC	FTO decreased, inhibit MMP‐9 mRNA and protein, lowering pro‐BDNF and m‐BDNF, leading to allodynia, anxiety and depression behaviors. Blocking FTO downregulation reverses above behaviors	Wang et al. [77]
Mice; 6‐week‐old; C57BL/6J	SNI	FTO, METTL14	PWT, PWL	SDH	FTO and CXCR3 up‐regulated, METTL14 down‐regulated. FTO silencing decreased the stability of *Cxcr3* mRNA, increased PWL and PWT, and decreased TNF‐α, IL‐1β, and IL‐6 levels. Overexpression of CXCR3 reversed the FTO silencing effect	Wu et al. [56]
Mice; 7–8 weeks old; male; C57BL/6J	Hemorrhage‐induced thalamic pain	FTO, YTHDF2	Mechanical, heat and cold tests	Ipsilateral thalamus	FTO increased, YTHDF2 decreased, the m^6^A site of *Tlr4* mRNA decreased, TLR4 mRNA and protein upregulated, and contralateral allodynia occurred	Fu et al. [85]
Mice; 6–8 weeks old; male; C57BL/6J	L4 SNL	FTO	Mechanical and thermal pain tests	L4 spinal cord	FTO binds to *Mmp24* mRNA, reducing its m^6^A modification and enhancing its translation, promoting ERK phosphorylation and NP	Ma et al. [84]
Rats; male; SD	L5 SNL	FTO	Mechanical, heat, and cold tests	L5 DRG, L5 SDH	Inhibit FTO decreasing mechanical allodynia, heat and cold hyperalgesia, and spontaneous pain	Zheng et al. [55]
Rats; 2–3 months old; male; SD	L5 SNL, CCI	FTO, YTHDF2	Mechanical, heat, and cold tests	L5 DRG	FTO increased, reducing m^6^A modification of *Ehmt2* mRNA and increasing G9a protein expression, suppressing MOR transcription and enhances neuronal excitability and allodynia	Li et al. [33]

Abbreviations: ACC, anterior cingulate cortex; CCI, chronic constriction injury; DRG, dorsal root ganglia; ERK, extracellular signal‐regulated kinase; FTO, fat‐mass and obesity‐associated proteins; IL, interleukin; m^6^A, N6‐methyladenosine; MOR, μ opioid receptors; NP, neuropathic pain; PWL, paw withdrawal latency; PWT, the paw withdrawal threshold; SD, Sprague–Dawley; SDH, spinal dorsal horn; SNI, spared nerve injury; SNL, spinal nerve ligation; TNF‐α, tumor necrosis factor‐α.

## 4 Molecular Mechanisms of Exercise‐Regulated m^6^A Modification

### 4.1 Impact of Exercise on FTO and m^6^A‐Modification Target Genes

Exercise can influence RNA methylation levels by regulating the expression and activity of m^6^A‐modifying enzymes. For instance, long‐term exercise may downregulate the expression of the demethylase FTO, leading to an increase in m^6^A‐modified transcripts in the hippocampus and hypothalamus. Thus, exercise may be an effective intervention strategy for epigenetic regulation [20]. Exercise also influences the expression of target genes associated with neurological disorders through m^6^A modification [94].

#### 4.1.1 Improvement in Anxiety and Synaptic Transmission

A 14‐day chronic restraint stress (CRS) model study in adult mice found that exercise restores m^6^A homeostasis in the medial prefrontal cortex (mPFC), enhances the m^6^A modification of excitatory synapse‐related genes, activates neurons projecting to the BLA, and increases the biosynthesis of the methyl donor SAM in the liver. These phenomena promote brain RNA methylation and neural activity, exerting anxiolytic effects [94]. CRS disrupts the metabolic regulation of epigenetic homeostasis in the brain, affecting cortical activity, whereas exercise restores cortical m^6^A levels by enhancing liver biosynthesis, thereby improving resilience against environmental stress [94]. This liver–brain axis mechanism reveals a novel pathway for exercise‐induced epigenetic regulation in response to stress.

#### 4.1.2 Improvement in Heart Failure

**FIGURE 3:**
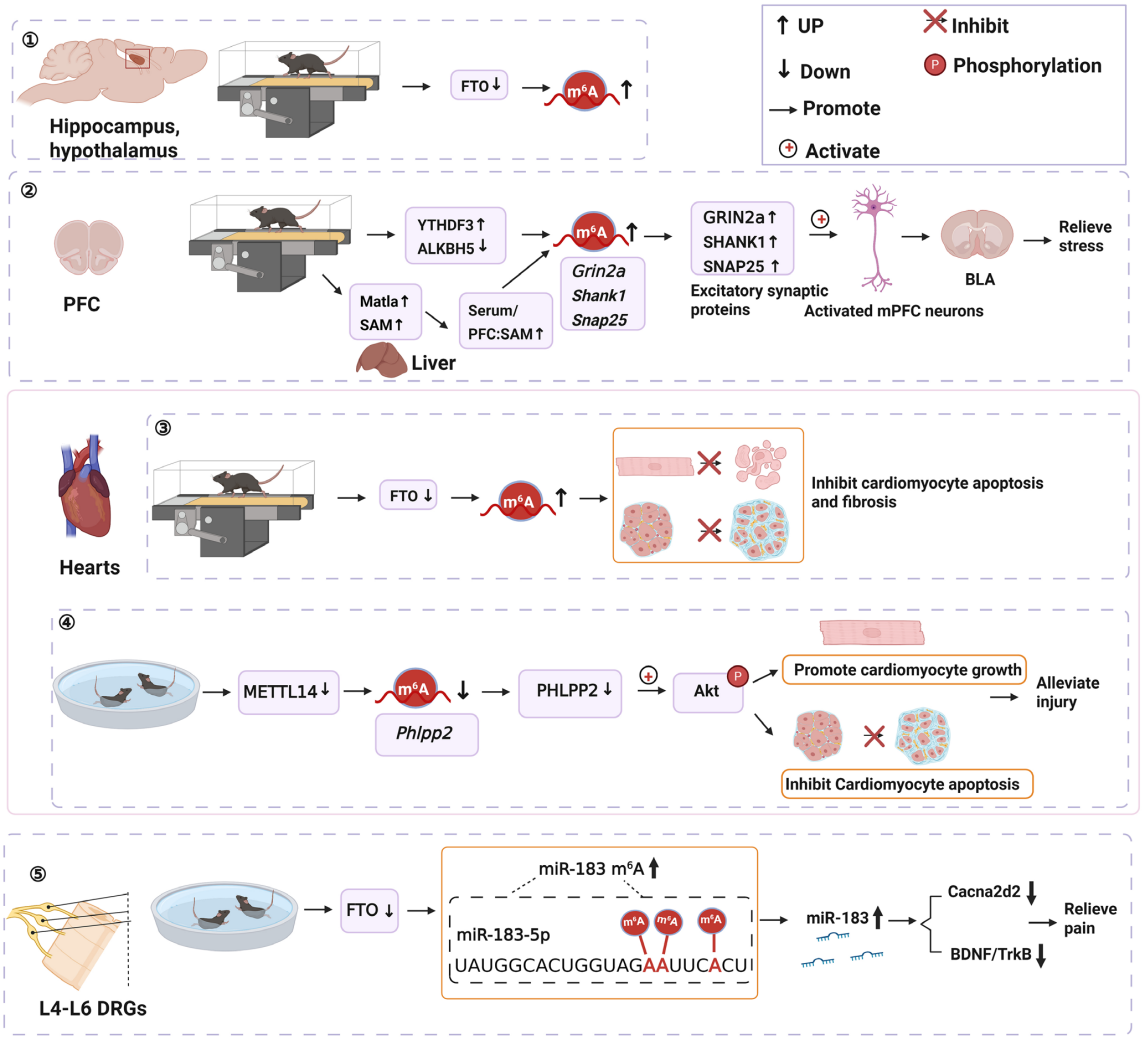
Effects of exercise‐regulated m^6^A modification on diseases in animal models. Akt, protein kinase B; BDNF, brain‐derived neurotrophic factor; BLA, basolateral amygdala; DRG, dorsal root ganglia; FTO, fat mass and obesity‐associated protein; m^6^A, N6‐methyladenosine; PFC, prefrontal cortex; SAM, S‐adenosylmethionine; TrkB, tropomyosin receptor kinase B.

Exercise also improves cardiac phenotypes in heart failure models by downregulating FTO and increasing m^6^A levels [19]. In heart failure with preserved ejection fraction (HFpEF) models, 8 weeks of moderate‐intensity exercise increases total m^6^A levels and downregulates FTO expression. FTO overexpression further counteracts the benefits of exercise by promoting cardiomyocyte apoptosis and fibrosis [19]. Additionally, aerobic exercise reduces m^6^A methylation levels in the hearts of high‐fat‐diet mice, modulating the expression profiles of m^6^A modifications in circular RNAs and thereby improving pathological cardiac remodeling [95]. Thus, exercise can prevent anxiety disorders induced by stress. Exercise induces significant downregulation of METTL14, which reduces its binding to m^6^A sites on the long noncoding RNA NEAT1 and suppresses NEAT1 expression [96]. Additionally, this study finds that compared with healthy controls, NEAT1 is upregulated in the plasma of patients with coronary heart disease (CHD). Compared with CHD patients, NEAT1 is downregulated in CHD patients who engage in regular exercise [96] (Table S1). These findings reveal how exercise influences the function of noncoding RNA through m^6^A modification, offering new insights and potential therapeutic targets. Endurance exercise also alleviates cardiac ischemia–reperfusion injury and adverse cardiac remodeling by downregulating the m^6^A methyltransferase METTL14 [97]. These studies demonstrate that exercise modulates m^6^A modification to influence the expression of target genes, thereby improving neurological and cardiovascular diseases. In summary, exercise improves synaptic transmission and exerts anxiolytic effects in the mPFC through m^6^A modification. By downregulating FTO and METTL14, exercise improves heart failure and ischemia–reperfusion injury, providing new therapeutic targets for cardiovascular diseases (Figure 3 and Table 3).

**TABLE 3:** Effects of exercise‐regulated m^6^A on diseases in animal models.

Species/age/sex/strain	Model	m^6^A regulatory factor	Exercise mode/protocols	Behavioral test	Sample	Main results	References
Mice; 6 weeks old; male; C57BL/6J	SNI	FTO	Swimming, moderate‐intensity, starting from postoperative Day 3, comprising 30 sessions. For the first 6 sessions, the duration was gradually increased from 30 to 50 min, and the remaining 24 sessions lasted 60 min/day, 6 weeks	MWT; cold allodynia response time	L4–L6 DRGs	Swimming exercise downregulated FTO mRNA and protein expressions, promoted the m^6^A modification of miR‐183, showed an association with the increased expression levels of miR‐183. Further reduced the downstream target gene CACNA2D2 and the BDNF/TrkB signaling pathway, which alleviated mechanical and cold allodynia	Zheng et al. [21]
Mice; 8 weeks old; male; C57BL/6J	Cardiac I/R model	YTHDF2	Swimming, starting from 10 min twice a day, with a 10‐min increase per day until reaching 90 min/day, 4 weeks	NA	Heart	Inhibition of YTHDF2 promoted exercise‐induced physiological cardiac hypertrophy and attenuates I/R remodeling	Xu et al. [95]
Mice; 6–8 weeks old; male; C57BL/6J	Atherosclerosis model (HFD)	METTL14	Treadmill, gradually increase the time and speed of exercise during the first week, from Week 2 to Week 12, 15 m/min, 60 min, 5 day/week, 12 weeks	NA	Hearts	Exercise down‐regulate METTL14, reducing its binding to m^6^A sites on NEAT1 and suppressing NEAT1 expression, attenuated NEAT1‐mediated pyroptosis, and slowing atherosclerosis progression	Yang et al. [96]
Mice; 8 weeks old; male; C57BL/6J	HFpEF	FTO	Treadmill, moderate‐intensity, 10° gradient for 10 m/min, 50 min, 5 times a week, 5 days, 8 weeks	Exercise exhaustion test	Hearts	Exercise increased total m^6^A level, down‐regulated FTO, and inhibited the apoptosis and fibrosis of cardiomyocytes	Liu et al. [17]
Mice; 8–9 weeks old; male; C57BL/6J	Cardiac I/R model	METTL14	Swimming, starting from 10 min twice daily, with a 10‐min increase each day until reaching to 90 min, 4 weeks	NA	Hearts	Exercise alleviated cardiac I/R injury and adverse cardiac remodeling by downregulating the METTL14. METTL14 silencing reduced m^6^A modification of Phlpp2 mRNA, activating the Akt‐S473 signaling pathway and regulating cardiomyocyte growth and apoptosis	Wang et al. [77]
Mice; 5 weeks old; male; C57BL/6J	CRS	ALKBH5, YTHDF3	Treadmill, 10 m/min, 1 h/day, 14 days	OFT; EPM; marble burying test; light/dark box test	PFC	Exercise restored m^6^A homeostasis in PFC, enhanced m^6^A modification of excitatory synapse‐related genes, activates neurons projecting to the BLA. Meanwhile increased SAM in the liver, promoting brain RNA methylation, neural activity, and exerting anxiolytic effects	Yan et al. [94]
Mice; 8 weeks old; male; C57BL/6J	Exercise group vs. sedentary group	FTO	Treadmill, 12 m/min, moderate‐intensity, VO_2_ max 75%, 60 min/day, 5 days/week, 12 weeks	NA	Hippocampus and hypothalamus	Exercise decreased FTO expression and increased m^6^A level	Liu et al. [70]

Abbreviations: BDNF, brain‐derived neurotrophic factor; BLA, basolateral amygdala; CRS, chronic stress restraint; DRG, dorsal root ganglia; EPM, elevated plus‐maze; FTO, Fat mass and obesity‐associated protein; HFD, high‐fat diet; HFpEF, Heart failure with preserved ejection fraction; I/R, ischemia–reperfusion; m^6^A, N6‐methyladenosine; MWT, mechanical withdrawal threshold; NA, not applicable; OFT, open field test; PFC, prefrontal cortex; SAM, S‐adenosylmethionine; SNI, spared nerve injury; TrkB, tropomyosin receptor kinase B.

To provide a systematic overview of exercise interventions cited in this review, we summarize the key parameters (modality, intensity, frequency, duration, and animal model) in Table 3. The most commonly used exercise modalities are treadmill running and swimming. Typical protocols involve moderate‐intensity exercise (e.g., 60 min/day, 5 days/week, for 4–12 weeks). Notable examples include 6 weeks of swimming (60 min/day, 6 days/week) in the SNI mouse model of NP [21]; 12 weeks of treadmill running (12 m/min, 60 min/day, 5 days/week) in healthy mice [20]; and 8 weeks of treadmill running (10 m/min, 50 min/day, 5 days/week) in a heart failure with preserved ejection fraction (HFpEF) mouse model [19]. It is important to note that direct evidence linking exercise to m^6^A modification in neurological disorders and NP remains limited, and most current data come from studies on cardiovascular or stress‐related models.

### 4.2 Mechanisms of Exercise‐Induced Improvement in Neurological Disorders by m^6^A Modification

The exercise‐induced modulation of FTO expression increases m^6^A levels in the hippocampus and hypothalamus [20]. By epigenetically regulating m^6^A marks on genes related to plasticity, exercise promotes synaptic plasticity and neuroregeneration. For instance, the m^6^A modification of BDNF mRNA influences neuronal apoptosis under stress [62] while enhancing memory reconsolidation [63]. BDNF/TrkB signaling critically regulates synaptic plasticity and pain hypersensitivity post‐injury by activating the MAPK/ERK and PI3K/Akt pathways in the DRG [98, 99, 100, 101]. Ultimately, exercise‐driven m^6^A remodeling facilitates axonal regeneration and functional recovery through coordinated epigenetic and neurotrophic mechanisms [19]. Physical exercise and cognitive training enhance synaptic plasticity, learning, and memory while reducing the risk of complex diseases like AD. In adult male mice, exercise and cognitive training improve synaptic plasticity and cognitive abilities in the next generation, mediated by sperm RNA (e.g., miR‐212/132) [102]. Exercise and acute restraint stress (RS) differentially regulate the transcription and epigenetic mechanisms of BDNF in the mouse hippocampus [103].

Emerging evidence suggests a mechanistic link where exercise‐induced downregulation of the m^6^A demethylase FTO leads to the activation of the neuroprotective BDNF/TrkB signaling pathway. Specifically, long‐term exercise training has been shown to decrease FTO expression in the hippocampus and hypothalamus, concomitantly increasing global m^6^A levels [19, 20]. As FTO is a known eraser of m^6^A marks on Bdnf mRNA, its downregulation by exercise is predicted to enhance the m^6^A modification of Bdnf transcripts. Therefore, exercise‐driven remodeling of m^6^A, particularly the FTO/BDNF/TrkB axis, represents a coordinated epigenetic mechanism [19]. In summary, exercise enhances neurological function through m^6^A modification, mediated by FTO regulation of hippocampal gene expression, and promotes synaptic plasticity by activating the BDNF/TrkB pathway.

### 4.3 Potential Mechanisms of the Exercise‐Induced Alleviation of NP

Existing research reveals that exercise may alleviate NP through the following mechanisms: sensory‐pathway remodeling and inflammation regulation. First, exercise targets sensory pathway remodeling by modulating neuronal excitability and synaptic plasticity [104, 105]. Second, exercise modulates glial‐cell activation, macrophage phenotypes, and inflammatory expression, exerting neuroprotective effects [104, 106, 107, 108, 109, 110]. Exercise reduces IL‐1β, TNF‐α, and IL‐6 [111] while enhancing autophagy via BDNF/AKT/mTOR signaling and promoting microglial M2 polarization to alleviate NP [112]. High‐intensity swimming engages the resolvin E1‐chemerin receptor 23 axes to reduce pain and neuroinflammation [113]. Notably, different exercise modalities demonstrate benefits. Swimming reduces β‐NGF and BDNF expression in neuroma models and activates RvE1 receptors in ischemic pain [113]. Treadmill exercise inhibits astrocytic C3 expression [114], whereas wheel running modulates DRG macrophages and correlates with improvements in pain behavior [115]. These interventions collectively inhibit glial activation, regulate inflammation, and restore neuronal homeostasis [105, 114]. Specifically, FTO has been shown to influence the expression of pro‐inflammatory cytokines by modulating the stability of inflammation‐related mRNAs. For instance, FTO knockdown destabilizes Cxcr3 mRNA, thereby lowering the levels of pro‐inflammatory cytokines such as IL‐1β, TNF‐α, and IL‐6 and alleviating pain [56]. Given that exercise downregulates FTO expression in the DRG [21], it is plausible that exercise may suppress neuroinflammation in NP through a similar FTO‐dependent mechanism, reducing the stability or translation of key inflammatory gene transcripts via altered m^6^A modification. This could represent a novel epigenetic axis linking exercise to the resolution of neuroinflammation in NP.

Our recent preclinical study elucidates that swimming exercise alleviates neuropathic pain by modulating the m^6^A RNA methylation pathway [21]. Specifically, exercise downregulates FTO expression in the DRG, consequently elevating global m^6^A levels. This reduction in FTO enhances the m^6^A modification of miR‐183, leading to its upregulated expression. The elevated miR‐183, in turn, represses key pain‐associated targets, including Cacna2d2 (which encodes the CaVα2δ‐2 subunit of voltage‐gated calcium channels) and the BDNF/TrkB signaling pathway. Ultimately, this exercise‐induced FTO/m^6^A/miR‐183 axis attenuates neuronal hyperexcitability and neuroinflammation, thereby mitigating mechanical allodynia and cold hypersensitivity [21]. In summary, exercise offers neuroprotective benefits by inhibiting glial activation, regulating macrophage phenotypes, and restoring neuronal excitability. Whether m^6^A modification plays a role in the process of exercise alleviating NP is still unclear. Nevertheless, based on the role of FTO‐regulated m^6^A modification in NP, the effect of exercise on NP, and the role of exercise‐regulated m^6^A modification in other diseases, we speculate that exercise may affect the m^6^A modification of target genes by regulating FTO, thereby alleviating NP. Further laboratory investigation is required to clarify this assumption and the underlying mechanism.

## 5 Future Prospects

Epigenetic markers hold broad application prospects in the diagnosis, treatment, and prognosis of neurological disorders. Mutations in the FTO gene, particularly in intron 2, are associated with the development of AD [60]. Loss‐of‐function mutations in the FTO gene are associated with neurodevelopmental disorders [31, 65, 66] and may provide new therapeutic targets. In PD, METTL3 and FTO show no significant correlation with PD risk; however, reduced m^6^A levels and elevated FTO expression in the striatum of PD patients suggest a potential role for FTO‐regulated m^6^A modification in PD [11]. Future efforts should aim to investigate whether FTO could serve as a diagnostic and prognostic epigenetic marker for neurological disorders and NP. Additionally, targeting FTO with specific inhibitors or activators may provide novel therapeutic strategies. Our recent work, which delineates the swimming exercise/FTO/miR‐183/Cacna2d2 pathway in NP [21], serves as a foundational step in this direction.

In terms of clinical translation, several reliable methods have been established for detecting FTO/m^6^A‐related biomarkers. Methylated RNA immunoprecipitation sequencing (MeRIP‐seq) is widely used for transcriptome‐wide m^6^A profiling. Liquid chromatography–tandem mass spectrometry (LC–MS/MS) enables absolute quantification of global m^6^A levels. These techniques provide a foundation for clinical detection of FTO/m^6^A biomarkers. Liu et al. [12] found that FTO expression was significantly downregulated in the hippocampus of major depressive disorder patients compared to the healthy controls. Nevertheless, multiple challenges remain for clinical practice. First, sample accessibility is limited because most current studies detect FTO/m^6^A in brain tissues or DRG, which are difficult to obtain from living patients. Second, detection standardization is insufficient; inconsistent preprocessing, sequencing platforms, and analytical pipelines across laboratories lead to poor comparability of results. Third, the specificity and sensitivity of FTO/m^6^A as biomarkers require further validation, as abnormal FTO expression and disordered m^6^A modification are also observed in other systemic diseases. Moreover, their diagnostic value for early stage neurological disorders and NP remains to be verified.

Future investigations should prioritize several key areas. First, determining whether this FTO‐dependent mechanism generalizes to other exercise modalities (e.g., treadmill running and resistance training) and to other neurological disorders such as AD and PD. Second, exploring the temporal dynamics of exercise‐induced FTO downregulation and m^6^A remodeling will help identify optimal interventional time windows. Third, translating these findings from rodent models to human populations, potentially by examining FTO and m^6^A‐related biomarkers in patient cohorts undergoing exercise rehabilitation, will be crucial for clinical application. Finally, unbiased approaches like single‐cell RNA sequencing of DRG and brain regions post‐exercise could unveil broader, FTO‐independent epigenetic networks engaged by physical activity.

## 6 Conclusion

In the NP pathways, including the thalamus, spinal cord, and DRG, FTO‐regulated m^6^A modifications are involved in the pathological processes of NP, suggesting that FTO may be a potentially effective target for managing NP. In neurological disorders such as AD, PD, and depression, the critical role of FTO‐regulated m^6^A modifications has been identified in the hippocampus and striatum. The common mechanisms can be summarized as related to the regulation of downstream mTOR and BDNF/TrkB signaling pathways, as well as the modulation of neuronal excitability and synaptic plasticity. Additionally, studies have found that exercise can reduce FTO levels in the hippocampus and heart. The mechanism by which exercise regulates m^6^A modifications is associated with promoting synaptic plasticity, modulating neuronal excitability, and providing neuroprotection. Preliminary clinical evidence supports FTO as a potential biomarker, as FTO is downregulated in the hippocampus of patients with depression. However, current evidence is mainly derived from small‐scale samples, and standardized detection methods in peripheral blood or cerebrospinal fluid have yet to be established. We hypothesize that exercise may regulate neurological disorders and NP through FTO‐regulated m^6^A modification, and FTO has the potential to serve as a biomarker, but this requires further laboratory and clinical validation. Current evidence is mainly derived from small‐scale samples, and standardized detection methods in peripheral blood or cerebrospinal fluid have yet to be established.

## Author Contributions

Yanan Zheng wrote the main manuscript text and prepared figures and tables. Yili Zheng supported the revision. Peijie Chen and Xueqiang Wang supported the supervision. All authors reviewed the manuscript and approved the submission.

## Funding

This study was supported by grants from the National Natural Science Foundation of China (82372578).

## Ethics Statement

The authors have nothing to report.

## Consent

The authors have nothing to report.

## Conflicts of Interest

The authors declare no conflicts of interest.

## Supporting information


**Table S1:** Effects of m^6^A‐related biomarkers on human diseases.

## Data Availability

Data are available from the corresponding author upon reasonable request.
