# Supplementary material for: Exercise, Demethylase FTO, Neurological Disorders, and Neuropathic Pain: Potential Molecular Mechanisms
Source: CNS Neurosci Ther. 2026 Jul 27;32(7):e71051. doi: 10.1002/cns.71051 (PMC13403481; doi:10.1002/cns.71051)
Supplement: Supplementary file 1 — Table S1: Effects of m6A‐related biomarkers on human diseases. [file CNS-32-e71051-s001.docx]

**Abbreviations list:**

ACC: anterior cingulate cortex;

AD: Alzheimer disease;

Al(mal): aluminum-maltolate;

BDNF: brain-derived neurotrophic factor;

BLA: basolateral amygdala;

CCI: chronic constriction injury;

CCL: chronic constant light exposure;

CHD: coronary heart disease;

CRS: chronic stress restraint;

DRG: dorsal root ganglia;

ERK: extracellular signal-regulated kinase;

FTO: fat-mass and obesity-associated proteins;

HFpEF: Heart failure with preserved ejection fraction;

IL: interleukin;

m^6^A: N6-methyladenosine;

MOR: μ opioid receptors;

mTOR: mammalian target of rapamycin;

NMDAR: N-methyl-D-aspartate receptor;

NP: neuropathic pain;

PD: Parkinson's disease;

PFC: prefrontal cortex;

SAM: S-adenosylmethionine;

SDH: spinal dorsal horn;

SNI: spared nerve injury;

SNL: spinal nerve ligation;

TNF-α: tumor necrosis factor-α.

TrkB: Tropomyosin receptor kinase B;

TSC1: tuberous sclerosis complex;

UTR: untranslated regions.

**Table S1. Effects of m^6^A-related biomarkers on human diseases**

| Participants/group/age/sex | m^6^A regulatory factor | Exercise protocols | Evaluation indicators | Sample | Main results | Reference |
| --- | --- | --- | --- | --- | --- | --- |
| 1. CHD (60 males and 50 females; 54.24±5.55 years old); 2. CHD patients with regular exercise habits (40 males and 30 females; 54.17±7.61 years old); 3. 69 healthy controls. | NA | CHD patients with regular exercise habits  performed long-distance walking, 60 min, 5 days a week. | NA | Plasma | Compared with healthy control, NEAT1 was upregulated in the CHD patients. Compared with CHD patients, NEAT1 was downregulated in CHD patients with regular exercise. Also found that exercise reduces m^6^A sites on the NEAT1 of mice’s hearts. | Yang et al., 2023[1] |
|  |  |  |  |  |  |  |
| 1. MDD patients (3 males and 6 females); 2. healthy individuals (4 males and 5 females); 18-40 years old | The methyl- donor SAM | NA | HAMD; HAMA | Serum | The serum SAM level of the patients was decreased, which was negatively correlated with the HAMA score. | Yan et al., 2022[2] |
|  |  |  |  |  |  |  |
| (1) MDD patients (20 females and 16 males); 39.9 ± 12.5 years old;  (2) 20 healthy controls (10 females and 10 males); 36.6 ± 11.1 years old | FTO | NA | NA | Hippocampus | FTO is downregulated in the hippocampus of MDD patients. | Liu et al., 2021[3] |

Abbreviations: CHD: coronary heart disease; CRS: chronic stress restraint; EPM: elevated plus-maze; HAMD: Hamilton Depression Scale; HAMA: Hamilton Anxiety Scale; MDD: major depressive disorder; NA: not applicable; OFT: open field test; SAM: S-adenosyl methionine.

***Reference***

1. Yang Q, Chen S, Wang X, Yang X, Chen L, Huang T, Zheng Y, Zheng X, Wu X, Sun Y and Wu J (2023) Exercise Mitigates Endothelial Pyroptosis and Atherosclerosis by Downregulating NEAT1 Through N6-Methyladenosine Modifications. Arterioscler Thromb Vasc Biol 43:910-926. <http://doi.org/10.1161/atvbaha.123.319251>

2. Yan L, Wei JA, Yang F, Wang M, Wang S, Cheng T, Liu X, Jia Y, So KF and Zhang L (2022) Physical Exercise Prevented Stress-Induced Anxiety via Improving Brain RNA Methylation. Adv Sci (Weinh) 9:e2105731. <http://doi.org/10.1002/advs.202105731>

3. Liu S, Xiu J, Zhu C, Meng K, Li C, Han R, Du T, Li L, Xu L, Liu R, Zhu W, Shen Y and Xu Q (2021) Fat mass and obesity-associated protein regulates RNA methylation associated with depression-like behavior in mice. Nat Commun 12:6937. <http://doi.org/10.1038/s41467-021-27044-7>
